# NF-κB Signaling in Macrophages: Dynamics, Crosstalk, and Signal Integration

**DOI:** 10.3389/fimmu.2019.00705

**Published:** 2019-04-09

**Authors:** Michael G. Dorrington, Iain D. C. Fraser

**Affiliations:** Signaling Systems Section, Laboratory of Immune System Biology, NIAID, DIR, NIH, Bethesda, MD, United States

**Keywords:** NF-κB, macrophages, innate immunity, cell signaling, technologies

## Abstract

The nuclear factor-κB (NF-κB) signaling pathway is one of the best understood immune-related pathways thanks to almost four decades of intense research. NF-κB signaling is activated by numerous discrete stimuli and is a master regulator of the inflammatory response to pathogens and cancerous cells, as well as a key regulator of autoimmune diseases. In this regard, the role of NF-κB signaling in immunity is not unlike that of the macrophage. The dynamics by which NF-κB proteins shuttle between the cytoplasm and the nucleus to initiate transcription have been studied rigorously in fibroblasts and other non-hematopoietic cells, but many questions remain as to how current models of NF-κB signaling and dynamics can be translated to innate immune cells such as macrophages. In this review, we will present recent research on the dynamics of NF-κB signaling and focus especially on how these dynamics vary in different cell types, while discussing why these characteristics may be important. We will end by looking ahead to how new techniques and technologies should allow us to analyze these signaling processes with greater clarity, bringing us closer to a more complete understanding of inflammatory transcription factor dynamics and how different cellular contexts might allow for appropriate control of innate immune responses.

## Introduction

Transcriptional regulation of gene expression provides the basis for the responsiveness of cells to external stimuli such as changing microenvironment, infectious interlopers, or chemokine gradients. The bridge between stimulation and transcription is formed by a complex network of signaling pathways that work to activate transcription factors, which translocate to the nucleus and initiate discrete transcriptional programs. In the thirty-plus years since its discovery by Sen and Baltimore (1), few (if any) inducible signaling pathways have been studied in greater detail than that of nuclear factor-κB (NF-κB). First discovered in human B cells, it was quickly discovered that NF-κB is expressed in nearly all cells across the animal kingdom, dating back to invertebrates (2) and jawless fish (3). First shown to regulate the expression of the κ light-chain of antibodies in B cells, NF-κB was soon found to regulate an enormous range of genes in varying cell types and contexts, opening up an exciting new era in the study of signaling pathways driving gene transcription (4).

NF-κB signaling is crucial for a multitude of important immunological transcriptional programs, including inflammatory responses to microbes and viruses by innate immune cells (2, 5, 6), development and activation of adaptive immune cells (7, 8), as well as the development of secondary lymphoid organs (9). In this review, we will focus on the innate immune aspects of NF-κB signaling, especially in the mononuclear myeloid cell compartment, where NF-κB regulates thousands of primary and secondary response genes including cytokines, chemokines, transcription factors, antimicrobial peptides, and interferon (IFN)-stimulated genes (ISGs) (10–14). While NF-κB gene knock-out (KO) studies, next-generation sequencing, and advances in computational biology have provided us with a wealth of information regarding the transcriptional *outcomes* of NF-κB signaling, there is still much to be learned about the signaling process itself, which is complicated by cell type-, tissue-, and stimulus-specific variability in signaling components and their spatio-temporal dynamics.

With this review, we aim to outline recent work on the dynamics of NF-κB signaling in macrophages and other innate immune cells, with an emphasis on pattern recognition receptor (PRR) stimulation. First, we will describe the key findings of the many studies on NF-κB signaling in cell-free conditions and in non-immune cells such as fibroblasts and epithelial cells so as to contrast these with macrophage-based studies. We will also touch on how crosstalk between NF-κB and other signaling pathways, thresholding of pathway activation, and feedback loops can modulate the inflammatory response in macrophages by altering NF-κB activation. We will then cover the much smaller body of research on NF-κB signaling in other innate immune cells before discussing new tools that are being used to gain better spatio-temporal resolution of the NF-κB pathway, including novel reporter-based assays and their use in the ever-expanding field of computational modeling.

## NF-κB Signaling Dynamics

The NF-κB signaling module consists of five NF-κB monomers (RelA/p65, RelB, cRel, NF-κB1 p50, and NF-κB2 p52) which can dimerize to form up to 15 unique transcription factors and interact with the κB consensus motif found in many gene promoters, as well as five inhibitory proteins (IκBα, β, ε, γ, and δ) that make up the IκB protein family. Although the specific DNA sequence that constitutes a κB site is quite broadly defined, sites associated with individual genes have been shown to be highly evolutionarily conserved (15). Furthermore, unique NF-κB dimers can induce disparate transcriptional responses based on differences in these κB sequences that are as small as one nucleotide (16). This should be considered when evaluating the comparative functions of different NF-κB dimers activated concurrently in the same cell, as certain sites may preferentially bind specific dimers. Unlike the other NF-κB Rel proteins, NF-κB1 p50 and NF-κB2 p 52 are translated as precursor proteins (p105 and p100) that are autoinhibited by their c-terminal domains (also known as IκBγ and IκBδ, respectively), which are homologous to the “professional” IκB proteins (17). In their processed forms, p50 and p52 can form homodimers which lack the transcriptional transactivation domain present in the Rel proteins and can thus function in an inhibitory capacity. A subset of the NF-κB and IkB proteins are constitutively expressed in all mammalian cell types, including erythrocytes (18), with the activity of the NF-κB dimers inhibited at low levels of pathway activation through binding to one of the IκB proteins. The IκB proteins inhibit NF-κB transcription by occluding DNA-binding sites on the Rel proteins and preventing the translocation of the bulk of NF-κB dimers into the nucleus, resulting in only small amounts of inactive NF-κB trafficking between the nucleus and cytoplasm periodically (19). The particular dimer combinations present in a given cell are dependent on multiple factors, including cell type and tissue environment, which likely contribute to differential outcomes of NF-κB signaling depending on context.

Along with diversity in dimer repertoire, NF-κB signaling is also governed by two separate activation strategies, known as “canonical” and “non-canonical” signaling. Non-canonical NF-κB signaling occurs upon stimulation of a subset of the tumor necrosis factor superfamily receptors (TNFRs), and is slower and longer-lasting than canonical signaling. In this case, stimulation leads to the proteolytic processing of the p100 precursor protein into its active form p52, releasing it from auto-inhibition and leading to transcriptional activation by p52:RelB dimers. As such, these two NF-κB proteins are often termed “non-canonical NF-κBs” (7). While non-canonical signaling is important (especially in lymphoid organ development), for the bulk of this review we will focus on canonical signaling, as this is the primary pathway initiated after the ligation of either inflammatory cytokine receptors or PRRs. The canonical NF-κB response is also much faster than non-canonical signaling (20), making this pathway especially important during innate immune responses in first-responder cells such as macrophages.

### Canonical NF-κB Signaling

**Figure 1:**
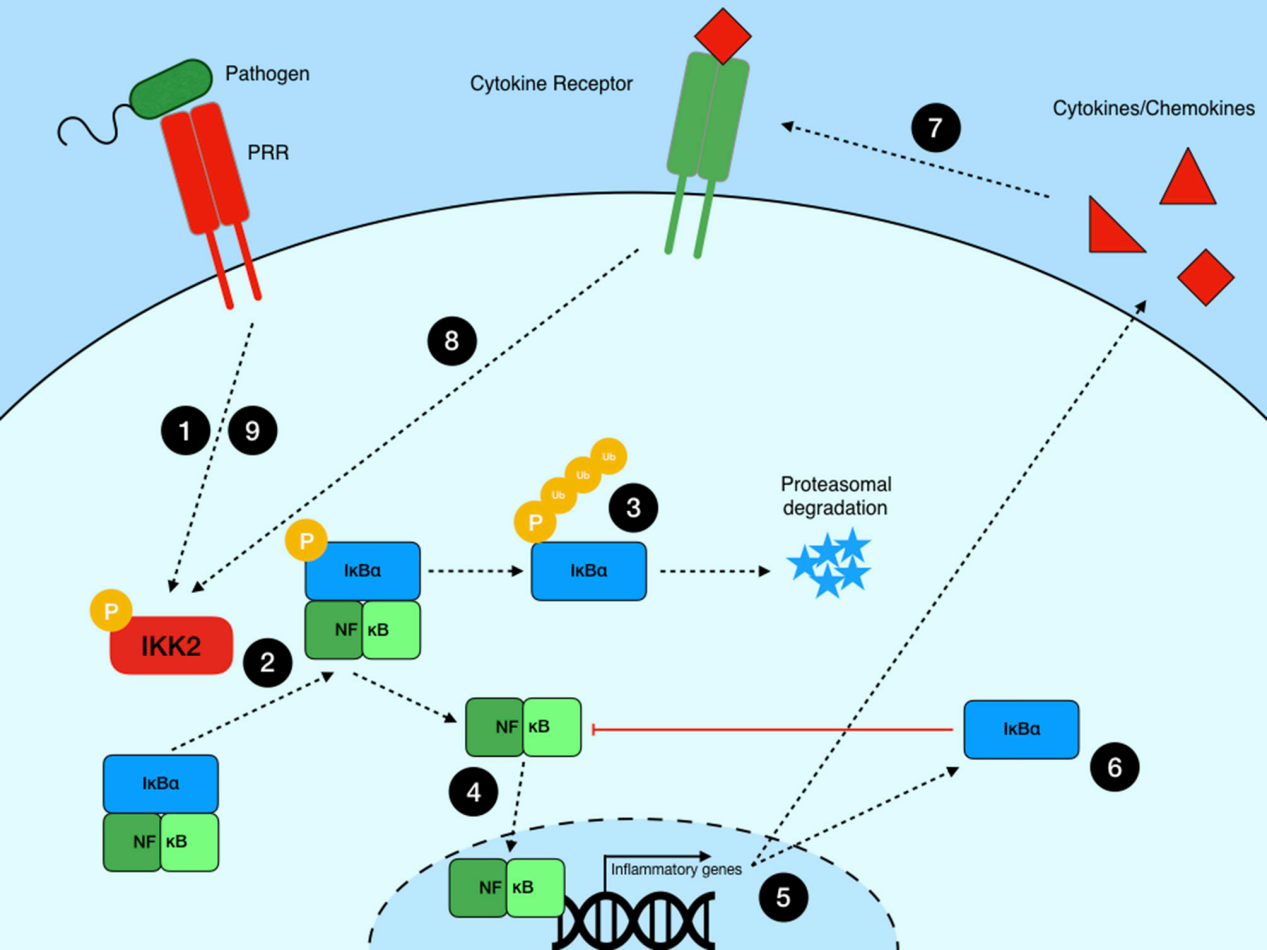
Canonical NF-κB signaling pathway. NF-κB signaling is initiated when a PRR or cytokine receptor recognizes its ligand, starting a signaling cascade (1) that converges on the phosphorylation of the IKK2 complex. IKK2 then phosphorylates IκBα (2), leading to its polyubiquitination and (3) subsequent degradation by the proteasome. This releases NF-κB dimers from negative regulation and (4) allows them to translocate to the nucleus to (5) initiate inflammatory gene transcription. (6) *De novo* synthesis of IκBα acts as a negative regulator of NF-κB-dependent transcription, limiting inflammation in the absence of further signaling events. (7) Primary response genes include those encoding cytokines such as TNF. (8) Release of these proteins leads to autocrine signaling through cytokine receptors. This, or (9) continued PRR ligation, create a positive feedback loop wherein NF-κB is periodically activated until these signals are eliminated.

Studies in non-hematopoietic cells have provided a strong foundation of information for our understanding of canonical NF-κB signaling (Figure 1). Cytokine or PRR stimulation and signal transduction result in the phosphorylation of the IKK2 complex, which is made up of IKKβ and NF-κB essential modulator (NEMO, also known as IKKγ). The activated IKKβ then phosphorylates IκB with NEMO acting as a scaffold (21), leading to tagging of IκB for degradation via K48-linked ubiquitin chains by the F-box-containing E3 ligase β-TrCP [comprehensively reviewed in Kanarek and Ben-Neriah (22)]. IκB is then degraded by the proteasome, leaving the NF-κB dimer free to translocate to the nucleus and initiate the transcription of primary response genes such as *TNF* and *IL1B*.

While seemingly simple, this classic NF-κB activation cycle can lead to countless variations in gene expression depending on a host of factors. The first level of complexity in NF-κB signaling arises from the multitude of ligands and receptors that stimulate this pathway. For example, NF-κB activation can arise through the stimulation of many cytokine receptors like TNFRs and IL1Rs (23), PRRs such as TLRs (24), MAVS (25), STING (26), and NOD-like receptors (27), as well as T and B cell receptors (28, 29), among many others. While macrophages do not express all of these receptors (e.g., T and B cell receptors), there remain numerous ways in which NF-κB can be stimulated in these cells. For the purpose of brevity, we will focus mostly on PRRs such as TLRs when discussing NF-κB signaling in macrophages. It should be noted however that the question of how variable NF-κB dynamics in response to a wide range of input pathways (often activated at different times during pathogen infection with a multi-PAMP microbe) contribute to transcriptional outcomes in the same cell type, remains an important topic for future study.

While the ligation of each of the receptors mentioned above can directly activate NF-κB signaling, they also feed into other signaling pathways that can interact, directly or indirectly, with NF-κB pathway components. One of the best studied examples of this regards the stimulation of TLR4 with lipopolysaccharide (LPS). TLR4 signaling through the adaptor molecule MyD88 leads to the activation of NF-κB, mitogen-activated protein kinase (MAPK), and IRF5 pathways (30), while subsequent endosomal TLR4 signaling through TIR-domain-containing adaptor protein inducing interferon-β (TRIF) leads to the activation of IRF3, and production of type I interferons (IFNs) and other antiviral genes (31). Therefore, *at least* four major signaling pathways are being activated by one stimulus. However, this does not take autocrine signaling through cytokine receptors into account. For example, triggering the TRIF-IRF3-IFN pathway leads to the release of type I IFNs, which will then bind to the type I IFN receptor (IFNAR) on the surface of the same cell (32), modulating signaling events that are still happening due to the original response to LPS or other cytokines (33). Therefore, there is the potential for considerable signal crosstalk after a cell is stimulated with one molecule. Needless to say, these interactions need to be considered when attempting to dissect seemingly “simple” pathways like canonical NF-κB signaling.

A recent study in human nasopharyngeal epithelial cells showed that the DNA binding sites of an NF-κB dimer are highly stimulus-specific (34). This work demonstrated that stimulation of epithelial cells with four common stimuli [LPS, TNF, Pam2CSK4, and Poly(I:C)] led to four significantly different patterns of RelA binding. Interestingly, the genes where RelA binding was enriched after stimulation with poly(I:C), a double-stranded RNA analog, were associated with the antiviral program. This shows that a stimulus from a specific class of pathogen can lead to a response tailored to that pathogen, even though RelA activation itself may appear to be stimulus-neutral in terms of IκB degradation and RelA nuclear entry. It will be interesting to see if future studies can determine the mechanism by which NF-κB binding sites are altered in a stimulus-specific manner. One possibility is that other stimulus-specific transcription factors activated alongside NF-κB act to augment or inhibit NF-κB chromatin binding and transcription at particular loci.

Stimulus identity is not the only complicating factor in the outcome of NF-κB signaling. Negative regulation of NF-κB signaling through synthesis of new IκB proteins and subsequent re-activation of the pathway can lead to a periodic oscillation of active NF-κB translocation to the nucleus, modulating subsequent gene expression dynamics. Because IκB genes are primary response gene targets of NF-κB transcriptional activity, they represent a powerful negative feedback loop (Figure 1). Newly synthesized IκB proteins bind to active NF-κB dimers, removing them from DNA binding and shuttling them back to the cytoplasm, where the complex can be reactivated and IκB can again be ubiquitinated and degraded via the proteasome. Some of the earliest studies on NF-κB activation dynamics demonstrated that this activation and negative feedback cycle can lead to periodic oscillations if two criteria are met (35). Firstly, the strength of negative feedback must be sufficient to exceed a threshold to favor cytosolic NF-κB sequestration, and secondly, after the consequent reduction in IκB gene synthesis, the input signal to the IKK complex must be sufficient to initiate another round of IκB degradation. The period during which the stimulus is sensed by the cells can therefore have profound effects on the resultant transcriptional response (35–37). Moreover, even in the presence of continued stimulation, negative regulation via deubiquitinases like A20 can arrest signaling by deubiquitinating both components of the IKK complex (38) and important signaling proteins such as TRAF6 (39). Therefore, the balance of positive and negative feedback signals has a profound impact on the transcriptional outcome of NF-κB activation.

The period and amplitude of NF-κB nuclear-cytoplasmic oscillations has been linked to differential gene expression in fibroblasts and epithelial cell lines using single-cell imaging and transcriptome analyses (40–42). Cell lines lacking individual or multiple NF-κB and IκB genes have reinforced these conclusions and have formed the basis for computational models of feedback dynamics and their effects on gene expression (43). However, as discussed below, recent studies performed in immune cells challenged with traditional immune stimuli have shown that these dynamics can vary substantially in different contexts and even change at different times in the cell cycle (44), and models need to be adapted depending on the cell type and status, as well as stimulus identity.

## NF-κB Signaling in Macrophages

NF-κB signaling in macrophages follows many of the principles elucidated using fibroblasts and other non-hematopoietic cells, with some exceptions. Some of these differences are simple, such as the increased importance of c-Rel in NF-κB dimers in macrophages, whereas RelA/p50 dimers predominate in fibroblasts. For example, c-Rel is especially important for the transcription of *IL12B* (IL-12 p40) in macrophages (12), as well as the resolution of inflammation via the transcription of an enzyme important for melatonin synthesis (45). Additionally, mice lacking both c-Rel and p50 NF-κB proteins have impaired innate immune responses to bacterial sepsis, with macrophages being deficient in phagocytosis, bacterial killing, and antimicrobial peptide production (10). c-Rel is also a vital part of an NF-κB-ATF3-CEBPδ transcriptional circuit that allows macrophages to scale the inflammatory response based on transient vs. persistent TLR4 stimulation (46). This circuit prevents hyperinflammatory responses to relatively miniscule LPS challenges. It should also be noted, however, that not all macrophages are the same, even when it comes to the effects of particular NF-κB proteins on gene expression. For example, c-Rel activity has differential effects on gene expression depending on whether the macrophage cells are tissue-resident or elicited from the blood (11). Once again, the complete context within which the signaling is occurring must be considered when predicting the transcriptional outcomes of NF-κB signaling.

### NF-κB Dynamics

Much of the work described above provides a framework in which NF-κB signaling specificity can be encoded by the period and amplitude of NF-κB nuclear/cytosol oscillations upon TNF stimulation of fibroblasts. However, work in our lab and others has shown that NFκB oscillation is a relatively rare occurrence in macrophages stimulated with TLR ligands (41, 47–50). While oscillation is observed in a small proportion of cells (41), most LPS activated macrophages show a single, longer-lasting NF-κB nuclear translocation event. This is, in part, supported by a positive feedback loop wherein RelA drives its own transcription and favors sustained nuclear occupancy of NF-κB in an LPS dose-dependent manner (50). This positive feedback and sustained NF-κB nuclear occupancy may also be supported by cRel, which is also dose-dependently induced by LPS (46). The sustained nuclear occupancy of LPS-activated NF-κB can be correlated with target gene transcription, as demonstrated by analysis of single macrophage cells expressing both GFP-RelA and a TNF promoter-driven mCherry reporter (50). The critical role of the TRIF pathway in supporting LPS-driven TNF responses (14, 47–49, 51–54), is also reflected in the NF-κB nuclear dwell time as sustained nuclear NF-κB is diminished in TRIF-deficient cells (47–49).

The duration of NF-κB nuclear occupancy in activated phagocytes is also regulated by additional transcription factors. Unbiased genome-scale gene perturbation screens have identified a critical role for the transcription factor Ikaros in supporting both RelA positive feedback and TNF production (50, 55, 56). Sustained NF-κB chromatin binding was shown to be severely diminished in Ikaros-deficient cells (55), which brings up the possibility that not only do multiple transcription factor pathways converge to support sustained NF-κB nuclear activity after LPS challenge, but also that long-lasting NF-κB chromatin binding is necessary to integrate signals from a complex array of inputs onto a broad landscape of activated enhancers and promoters.

The complex relationship between NF-κB dynamics and transcriptional control in macrophages was recently investigated further by coupling the dynamics of fluorescently labeled RelA with single cell RNA-seq (41). In this study, cells were categorized based on either transcriptome analysis or NF-κB dynamics and it was found that a strong, long-lasting nuclear RelA signal correlated with increased expression of inflammatory cytokine genes, while oscillatory behavior was rare. This supports the concept that robust cytokine expression in macrophages requires sustained NF-κB nuclear occupancy. By analyzing single cells, the authors were also able to show that the behavior of individual cells did not necessarily reflect the population as a whole and that different modes of activation existed in the population. This echoes prior experiments showing that many of these distinct dynamics (even to the same stimulus), and their resulting effects on gene expression, are lost in population-based analyses (42).

**Figure 2:**
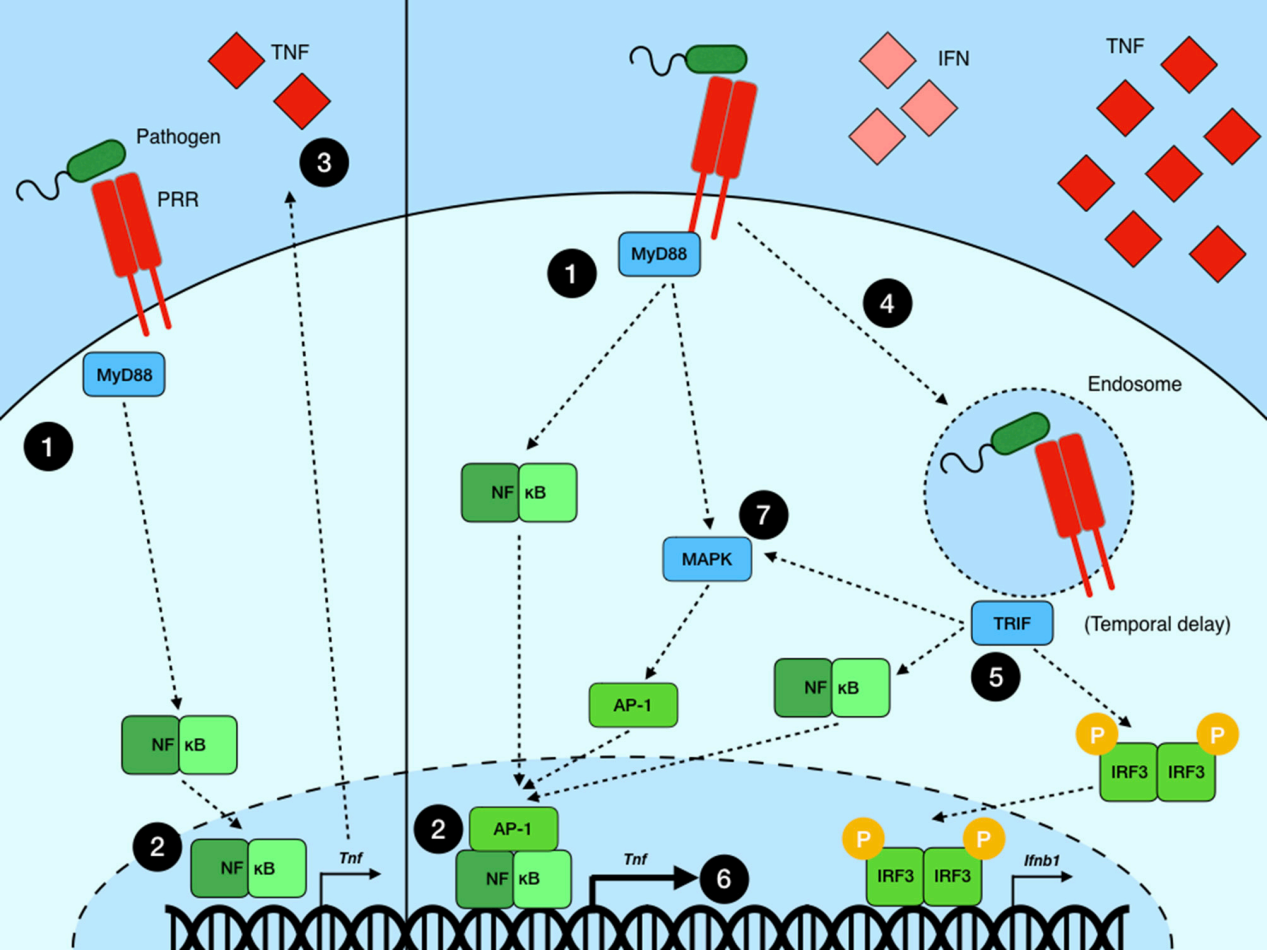
Signal crosstalk in NF-κB transcriptional regulation. **(Left)** An example of a simplified NF-κB signaling model wherein a pathogen is recognized by a PRR (e.g., TLR4), which (1) signals through MyD88 to (2) initiate NF-κB-mediated transcription and (3) subsequent cytokine production. **(Right)** A more accurate model of NF-κB signaling wherein a pathogen is recognized by a PRR (e.g., TLR4) on the surface of the cell, leading to (2) NF-κB activation via MyD88. (4) Subsequently, the pathogen is brought into a sub-cellular compartment where it is sensed by an alternate PRR (or, in the case of TLR4, the same PRR but in a new sub-cellular context) which signals through a second adaptor TRIF (5), leading to the activation of more NF-κB dimers as well as other transcription factors, such as IRF3, leading to type I IFN production, and AP-1, which is activated via the MAPK pathway. Upon translocation to the nucleus, these multiple transcription factors act to synergize or antagonize each other, more precisely tailoring the inflammatory response to the pathogen. (6) In this example, NF-κB and AP-1, each activated by multiple inputs, together produce significantly greater amounts of TNF than singly activated NF-κB on its own. (7) It should be noted that, while both MyD88 and TRIF are known to activate MAPKs, it remains unclear what their relative contributions are in regard to AP-1 activation and TNF production in the context of LPS stimulation.

While the amplitude of NF-κB translocation to the nucleus can be correlated with inflammatory gene induction and sustained expression, multiple recent studies hint at the importance of the integration of multiple signaling processes (47–49). Attempts at modeling NF-κB dynamics in macrophage cell lines stimulated with LPS show that both the MyD88 and TRIF signaling pathways are necessary for robust TNF production. Two of these studies argue that MyD88 is necessary for the initiation of *Tnf* transcription by activating NF-κB through the canonical pathway (47, 48). TRIF activation downstream of TLR4 signaling from the endosome then contributes to sustaining this response via the activation of the MAPKs p38 and Erk. These kinases subsequently act to stabilize TNF mRNA via the phosphorylation of MK2, as well as supporting the translation and secretion of TNF protein (47). Further modeling of MyD88- and TRIF-associated NF-κB activity showed again that MyD88 signaling is indispensable for initiating NF-κB shuttling to the nucleus while cell-to-cell variation in nuclear occupancy after the initial translocation depends primarily on TRIF activity (48). Whether IRF3 activation and nuclear occupancy downstream of TRIF signaling, or a separate arm of the TRIF pathway, support NF-κB dynamics remains elusive. However, a more recent study using dual TNF promoter and NF-κB reporters showed that, while initial NF-κB activation is independent of TRIF activation, *Tnf* promoter activity depends greatly on TRIF's involvement downstream of LPS stimulation (49). This suggests that TNF expression may require co-operation between NF-κB activation and that of AP-1, which is induced downstream of TRIF signaling through the MAPKs (57). One possibility is that the relative timing of MyD88- and TRIF-mediated signaling plays a critical role and that significant TNF production requires a delayed, but longer-lasting, TRIF-mediated signaling event leading to synergism between NF-κB- and AP-1-driven transcription of *Tnf* (Figure 2). All told, these studies suggest that while the dynamics of NF-κB translocation to the nucleus are important in determining the quality and quantity of the inflammatory response in pathogen-challenged macrophages, the overall outcome of NF-κB activation is substantially affected by crosstalk with other signaling pathways and transcription factors.

**Figure 3:**
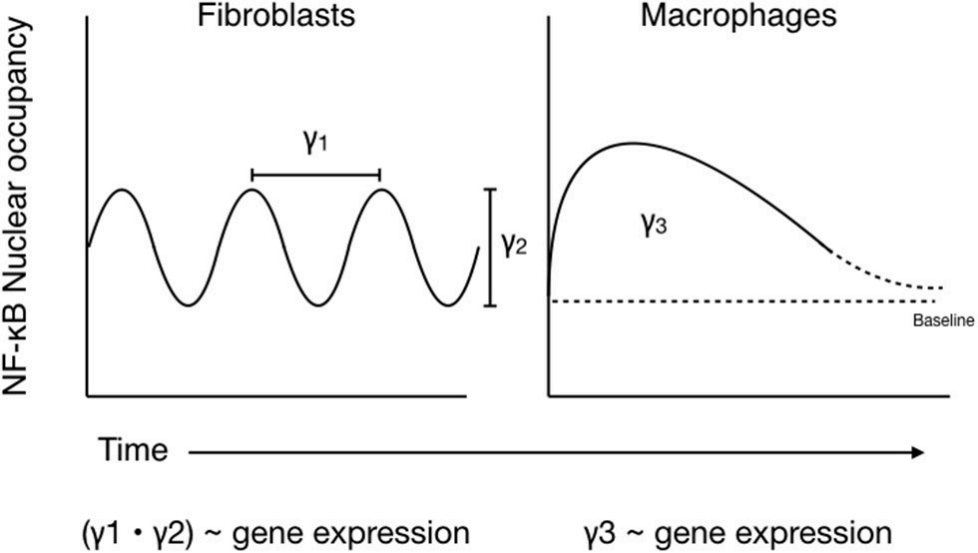
Comparing models of NF-κB translocation dynamics in macrophages vs. fibroblasts. ***(Left)*** NF-κB activation in fibroblasts is associated with periodic oscillations of NF-κB dimers between the nucleus, where they initiate transcription, and the cytoplasm, where they exist in complex with IκBα. Gene expression dynamics scale with the period (γ_1_) and amplitude (γ_2_) of these oscillations, which are influenced by variables such as signal strength, duration, and receptor identity. ***(Right)*** Conversely, NF-κB shuttling in macrophages is better represented by a single, strong nuclear translocation event which persists for as long as the stimulus remains and tends to remain above baseline for an extended period of time. As such, activation signals from additional pathways are likely integrated over this time, and gene expression dynamics correlate with area under the resultant nuclear NF-κB occupancy curve (γ_3_).

There has been a great deal of work performed in an attempt to accurately model NF-κB translocation dynamics in stimulated cells. Many of these models are based on the idea of recurrent NF-κB oscillations between the cytoplasm and nucleus, with the period and amplitude of the oscillations being linked to gene expression (37, 41, 58–60). The results of the above studies in macrophages, however, imply that these oscillations are rarer in innate immune cells than in non-hematopoietic cell models (41, 50). Perhaps a more accurate model for how NF-κB dynamics affect gene expression in macrophages would replace amplitude and period of oscillation with “height” and “width” of the single translocation peak observed in these cells. A further simplification of these would be to integrate the area under the curve when looking at RelA nuclear occupation over time (Figure 3). A possible benefit of this simplified model of NF-κB translocation could be that it is easier to integrate into more complex models of signaling involving the engagement of multiple pathways and transcription factors, as crosstalk among multiple pathways has a significant effect on gene expression.

Long-lasting nuclear occupancy of RelA increases the chances that significant numbers of NF-κB dimers are present in the nucleus when κB sites become available for binding, while also allowing time for other signaling pathways and transcription factors to modulate chromatin accessibility, tailoring the response to a particular stimulus or group of stimuli. For example, NF-κB must be present in the nucleus in order for the recruitment of the positive transcription elongation factor, p-TEFb, to primary response genes such as *TNF* (61). These genes are poised for transcriptional elongation before stimulation, but cannot be expressed as coding RNAs until they are bound sequentially by NF-κB, Brd4, and finally p-TEFb, releasing RNA polymerase II from its paused state (62). NF-κB and Brd4 also work together to establish super enhancers after TNF stimulation, profoundly altering the transcriptional landscape of the cell. In fact, chromatin binding of Brd4 at enhancer sites is nearly abolished when NF-κB activation is inhibited (63). It is possible that NF-κB translocation is extended in macrophages in part to facilitate these processes during acute immune events, though this has not been studied directly.

As we have mentioned, NF-κB dynamics vary greatly from cell-to-cell within a population, making single-cell analyses very important for analyzing how different signal kinetics affect transcriptional outcomes (42). Single-cell-based analyses in fibroblasts have shown that TNF stimulation leads to a quasi-digital response at the cellular level. Put simply, a cell either responds to the cytokine or not, with increasing dose leading to more cells being activated, accompanied by single-cell level increases in activation strength at higher concentrations of TNF (37, 42). However, studies in macrophages stimulated with LPS suggest a more analog phenotype, with almost all cells responding across a dose range and individual cell responses strengthening at higher ligand doses (41, 50, 64). This discrepancy has important implications in the macrophage response to infection wherein stimulus concentration is highly heterogeneous throughout a tissue. Analog responses that increase at the cellular level with ligand concentration may allow for increased tuning of an individual cell's response to its immediate environment, limiting bystander damage during inflammatory processes. This alteration of response thresholds will be discussed in further detail in the next section, but it is important to note that these thresholds will change based on NF-κB dynamics.

### Signaling Crosstalk

As mentioned above, one of the biggest complicating factors in NF-κB signaling outcomes is the fact that this pathway interacts, directly and indirectly, with a multitude of other signaling pathways during an immune response. As the predominant initiators of the inflammatory response to pathogens, macrophages must collect and integrate information, not only from multiple PRR pathways, but also from released host-derived mediators such as cytokines and interferons, leading to significant crosstalk among signaling pathways (Figure 2). In order to better simulate infectious stimuli, the outcome of stimulating macrophages with combined TLR ligands has been compared to single-ligand stimulations (65–68). These studies show that crosstalk between multiple TLRs simultaneously synergizes and antagonizes different gene subsets when compared to simply adding up the responses to individual TLR ligands alone. For example, stimulating macrophages with the TLR3 ligand poly(I:C) and the TLR7 ligand R848 together led to significantly greater production of IL-12p40 and IL-6 than that seen at saturating doses of either ligand alone (65, 69). This synergy only occurred when combining TLR ligands in such a way that both MyD88 and TRIF were utilized (i.e., triggering one TLR that signals through each adaptor), again showing that connecting these two pathways leads to enhanced inflammatory gene transcription likely by activating multiple transcription factor classes. Interestingly, work performed in fibroblasts stimulated with both TLR2 and TLR4 ligands showed that these cells make a signaling decision between the two pathways, depending on the relative dose of each of the ligands (53). This study did not record gene expression outputs, but rather looked at NF-κB shuttling dynamics, so it is not clear if dual-ligand stimulation leads to synergistic cytokine production in these cells. The studies mentioned here focus on combinatorial TLR stimulation but, as mentioned above, NF-κB stimulation is governed by much more than just TLRs, so further studies will be necessary to address how NF-κB signaling, and its outcomes, are altered by the co-stimulation of different classes of NF-κB-inducing receptors.

So far, we have only discussed how host signaling pathways interact to tailor the NF-κB-mediated immune response to a particular pathogen. However, this ignores the other side of the host-pathogen relationship. All pathogens have evolved ways in which to alter the immune response to allow for their survival and replication, and the NF-κB pathway is no exception. In fact, targeting immune signaling molecules via virulence factors is a very common strategy for pathogens as it allows them to alter receptor signaling without the need to target each receptor individually (70). For example, MyD88 and TRIF are common targets of pathogen-encoded proteases, preventing TLR signaling in infected cells (71–73). Other pathogens, such as pathogenic *E. coli* and *Clostridia spp*. target the NF-κB proteins themselves (74–77). Alterations in NF-κB signaling by pathogens have profound effects on the ability to mount an effective immune response, and the actions of pathogens on infected cells need to be considered when studying NF-κB signaling in these contexts. For more in-depth reviews of immune subversion by pathogens, please refer to Roy and Mocarski (78) and Hodgson and Wan (70).

Much like signal thresholding, explained below, the ability for macrophages to synthesize information from multiple stimuli in order to tailor the inflammatory response to a particular level of danger (in this case, a complex pathogen vs. a single stimulus) likely creates a balance between mounting an effective immune reaction and protecting the tissues from hyperinflammation.

### Thresholding

Placing thresholds on signaling inputs is an important determinant of the outcome of an inflammatory response. In order to assure the survival of a host, it is imperative that its immune system does not over-react to innocuous insult. For example, a small number of dead bacteria entering a wound should not elicit the same response as invasion of that wound by millions of live, replicating microbes. The dead bacteria can easily be disposed of by tissue-resident macrophages without the need for a full-blown inflammatory response, which could cause serious damage to the tissue. Similarly, the commensal relationship many bacteria have with higher organisms would be impossible were those hosts to mount a significant inflammatory response to otherwise benign microbes. As such, macrophages will not induce such a response unless they reach a particular threshold of signal density from their PRRs. This is supported by recent observations that different signaling outputs are disparately induced depending on the extracellular concentration of bacterial LPS (79). While NF-κB signaling was induced by very low concentrations of LPS (≤0.1 nM lipid A), many of the inflammatory genes associated with the NF-κB pathway, such as *Tnf*, were not transcribed. Only stimulation above a certain ligand threshold induced the bulk of NF-κB-related genes, which correlated with input from the MAPK pathway. Therefore, these two signaling pathways, which are stimulated via the same receptor, have separate thresholds of activation which will, in turn, affect the transcriptional outcome of both of their gene programs. Importantly, this demonstrates again that NF-κB signaling cannot be viewed in a vacuum, separate from other signaling events occurring within a cell.

There are likely many other factors affecting NF-κB activation thresholds in macrophages, including the activity of separate receptors. For example, stimulation of a macrophage with cytokines such as IFNs or TNF has profound effects on the stoichiometry of NF-κB pathway components and PRRs (33, 80), which will affect their relative availability to the signaling cascade. Inflammatory disease states (including aging), autoimmunity, and chronic infection will also alter signaling thresholds in macrophages, possibly exacerbating the underlying condition. In the case of aging, chronic stimulation of the NF-kB pathway leads to a shift in the inflammatory baseline of an individual, making it more difficult to mount an effective immune response against pathogens (81–83). The effects of aging on NF-κB processing and nuclear occupancy is an important topic for future investigation.

Thresholds will likely also be altered upon stimulation of a cell with a more complex ligand, such as a live bacterium, due to the cooperation of multiple signaling pathways. For example, it has been shown that signaling outcomes in macrophages are significantly altered when stimulated with live vs. dead bacteria (84–86). While these studies do not look specifically at NF-κB signaling, the effects of “vita-PAMPs” (or PAMPs that are only found in live pathogens) can be easily linked to alterations in signaling thresholds of common NF-κB read-outs. For example, bacterial mRNA, which is not found in dead bacteria preparations, is necessary for the activation of IRF3 and subsequent type I IFN responses (87). As mentioned above, IFN signaling can have a profound effect on NF-κB signaling outcomes. Vita-PAMPs are also vital for the stimulation of certain inflammasomes and the subsequent initiation of cell death (87), which also has important implications for NF-κB signaling, especially at the population level.

The threshold for activation of a macrophage also allows for sub-tissue microenvironments to limit immune damage. The probability that an individual macrophage reaches its threshold for signaling is directly proportional to the concentration of receptor ligand in its immediate vicinity (88). As such, cells that are further away from a focus of damage or infection are less likely to reach their inflammatory thresholds. If a small amount of ligand were capable of instigating a maximal inflammatory response from these cells, the immune-mediated damage to an infected tissue would be uncontrollable, leading to death of host tissues. Indeed, knocking out negative regulators of NF-κB signaling, such as the A20 deubiquitinase which deactivates the IKK complex, leads to lethal inflammatory diseases due to uncontrolled inflammation associated with TNFR signaling (89).

Finally, thresholds for a cellular response and the transcription of particular gene sets could also be dependent on the activation state of an individual unstimulated cell. It has been shown in HeLa cells that the amplitude and gene profile of a particular transcriptional response to TNF can be determined by the fold-change of nuclear NF-κB after stimulation, and not just on the total nuclear occupancy (90). It will be interesting to see how the nuclear NF-κB occupancy in a naïve macrophage may alter its signaling threshold.

## Other Innate Immune Cells

Reports on NF-κB signaling dynamics in other innate immune cells remain rare, though some work has been performed looking at cell-specific outcomes of NF-κB activation in neutrophils and dendritic cells, as briefly outlined here.

### Dendritic Cells

Dendritic cells (DCs) represent the bridge between innate and adaptive immune responses, presenting antigens to T and B cells in order to activate immunity to specific foreign invaders. The maturation of DCs into professional antigen presenting cells requires the NF-κB protein RelB (91). RelB-deficient DCs are unable to induce antigen-specific T cell responses both *in vitro* and *in vivo* (92). RelB-deficient mice suffer from spontaneous allergic airway inflammation, though the adoptive transfer of RelB+ DCs reverses this phenotype (93). RelB is considered a “non-canonical” NF-κB protein, however studies have shown that canonical stimuli and pathway components are essential for RelB activity in DCs (94, 95). RelB activity in DCs has been shown to be negatively regulated by the canonical IκB proteins IκBα and IκBε, and a canonical pathway activation mechanism is responsible for RelB-specific DC immune activity (91). Interestingly, this study also provided evidence that RelB may act as a downstream regulator of cRel. Whether cRel's importance in DCs is linked to their common lineage with macrophages remains to be seen, and how activation dynamics of the different Rel proteins in DCs compares to other cell types is another important topic for future investigation.

### Neutrophils

Neutrophils are tightly-regulated leukocytes that enter tissues during inflammatory responses in order to seek out and destroy pathogens. These highly inflammatory cells recognize PAMPs and subsequently activate NF-κB by the canonical pathway (96). In order to limit the inflammatory potential of neutrophils, they are very quick to undergo apoptosis after their activation. It has been shown that neutrophils have abnormally high levels of nuclear IκBα, which is responsible for dampening NF-κB-mediated gene expression and inducing apoptosis more quickly than in mononuclear cells (97). While this has not been tested directly, this study would suggest that NF-κB dynamics in neutrophils be characterized by cytosolic IκBα degradation, a single NF-κB translocation event, followed by re-synthesis of IκBα and subsequent apoptosis of the cell.

## The Future of NF-κB Signaling Pathway Analysis Is Bright

A common thread running through current signaling research is that we are now working in an era with greatly expanded capabilities in regards to analyzing smaller cellular populations in greater detail than ever before. To this end, new technologies are being harnessed in order to gain highly granular insights into signaling dynamics in single cells and in real-time. Signaling dynamics and their effects on gene regulation cannot be appropriately studied at the population level, nor with traditional time course experiments, so it is imperative that we keep harnessing new technologies to increase the resolution of these readouts. CRISPR-based gene manipulation, coupled with the single-cell transcriptional measurements and high-content imaging already in use, should permit more direct determinations of the causal relationships between NF-κB activation and transcriptional outcomes in the same single cell. Using these more accurate tools, single or multiple NF-κB, IκB, and IKK genes could be monitored or perturbed to better investigate how they interact to control gene expression in macrophages and other immune cells. This can also be extended to other modulators of this pathway, such as deubiquitinases and chromatin regulators. CRISPR can also be used to modify genes at their endogenous loci with fluorescently-tagged versions for imaging purposes. These fluorescent proteins can be tracked *in situ* to gain a better understanding of their dynamics through space and time, or to identify novel binding partners, filling in gaps in our understanding of pathway regulation. Using these techniques in multiple contexts (e.g., dose responses of ligand, multi-ligand stimulation, acute infection) will help us tease apart how signaling changes with these different parameters, creating a more complete model of NF-κB signaling responses and transcriptional regulation.

Another thing that is clear, based on the studies reviewed here, is that studying NF-κB requires the concurrent study of the many other signaling pathways that interact with it. Studying how and when RelA enters and exits the nucleus has provided us with a great deal of insight into the roles of this pathway, but has also uncovered the need to study additional signaling events simultaneously. To this end, the use of fluorescently-tagged reporter molecules will permit measurement of the temporal activity of multiple kinases, transcription factors, and gene promoters in a single cell (98). By increasing the amount of information we can capture from single cells, researchers can more accurately model the signaling dynamics of members of several signaling pathways concurrently. These models can then be used to better predict how cells will react to single or multiple stimuli or in different contexts. While we may never achieve a “unified theory of inflammation,” more accurate models of cell behavior will be a great benefit to drug discovery and personalized medical interventions.

Mathematical modeling of NF-κB activation and translocation dynamics is far more advanced than for most other signaling pathways, with some of the earliest models appearing almost 20 years ago (99). These models have improved over time as we have gained further understanding of the roles of the various NF-κB and IκB isoforms (100). Later, modeling studies combined with knock-outs for various NF-κB signaling proteins began to describe multiple feedback loops via multiple IκB proteins (101) as well as autocrine signaling through TNF receptors (14). Currently, models exist that encapsulate an enormous amount of information, including differences in NF-κB dimer identity, positive and negative feedback loops, and interactions between canonical and non-canonical signaling pathways (102–105). Unfortunately, there has been little published regarding NF-κB dynamic modeling in the past few years, despite the amount of data we have on NF-κB signaling increasing at pace. We hope that the current models of NF-κB-mediated responses will be updated to encapsulate these important studies expediently. Also, it will be important for future studies to integrate these models with other technologies like those described above so that we can see how applicable they are to *in vivo* systems. With a combination of mathematical modeling, genetic perturbation, and *in vivo* imaging, the power of these tools to predict transcriptional regulation, druggable targets, and the potential effects of genetic variation will increase exponentially.

Finally, due to the multitude of endogenous and exogenous factors that modulate NF-κB signaling in innate immune cells, it is vital that the effects of tissue microenvironments on inflammation are studied in much greater detail in the coming years. With the advent and growth of *in situ* genetic perturbations (106), multi-valent reporters (98), and *in vivo* imaging (107, 108), researchers can now, theoretically, look in real-time at signaling processes happening in animal models of inflammation and infection. Though there remain significant technical challenges in combining these technologies, the ability to look at specific cell populations *in vivo* will provide us with an enormous leap forward in how we understand signaling dynamics and their outcomes in truly relevant contexts, making the necessary investment worthwhile.

## Conclusions

NF-κB is a master regulator of innate immune responses, and vital to many of the roles that macrophages and other innate immune cells play in orchestrating the inflammatory response to pathogens. In this review, we have outlined the many variables that influence the outcomes of NF-κB signaling, including those that are cell-, tissue-, and stimulus-specific. Over 30 years of research has illuminated the dynamics of this signaling pathway and the genes that are regulated by it, leading to many breakthroughs in how we understand NF-κB function. However, much of this information has come from studying non-hematopoietic cells or pathway components in cell-free conditions. As new technologies and techniques have been developed over the past decade, it has become feasible to study NF-κB signaling in less tractable cell models such as primary macrophages as well as *in vivo*. Recent studies, outlined above, have highlighted differences in signaling dynamics in these contexts which act to support the goals of the innate immune system—that is, to regulate and tailor the inflammatory response to pathogens in order to balance the destruction of invaders with the limitation of potentially harmful hyperinflammation.

Macrophages are capable of integrating an impressive amount of information regarding the identity and virulence of pathogens, as well as endogenous cues present in their microenvironment, in order to modulate the immune response to best protect the host. Central to this ability are the many ways in which NF-κB signaling is modulated based on shifting thresholds of activation, the integration of information from various classes of PRR, and tight regulation of transcription through rigorous positive and negative feedback loops. How these components fit together in different contexts, and how we may be able to modulate or interfere with them to the benefit of patients, is an important field of future research.

The differences between inflammatory signaling in fibroblasts and macrophages (and other innate immune cells) allow for the host to survive most infectious threats, and it is important that we, as researchers, continue to study signaling in different contexts in order to gain a more thorough understanding of how these processes contribute to the immune response. By combining new technologies, we now have the ability to study these phenomena in greater resolution than ever before, even *in vivo* or in primary human cells. The future of NF-κB signaling research is bright—and perhaps fluorescent!

## Author Contributions

The writing strategy for this review was devised by MD and IF. The initial draft was written by MD with subsequent drafts edited by MD and IF collaboratively.

### Conflict of Interest Statement

The authors declare that the research was conducted in the absence of any commercial or financial relationships that could be construed as a potential conflict of interest.

## References

[1] Sen R Baltimore D. Inducibility of kappa immunoglobulin enhancer-binding protein Nf-kappa B by a posttranslational mechanism. Cell. (1986) 47:921–8. 10.1016/0092-8674(86)90807-X 3096580

[2] Hetru C Hoffmann JA. NF-kappaB in the immune response of *Drosophila*. Cold Spring Harb Perspect Biol. (2009) 1:a000232. 10.1101/cshperspect.a000232 20457557 PMC2882123

[3] Su P Liu X Pang Y Liu C Li R Zhang Q. The archaic roles of the lamprey NF-κB (lj-NF-κB) in innate immune responses. Mol Immunol. (2017) 92:21–7. 10.1016/j.molimm.2017.10.002 29031044

[4] Lenardo MJ Baltimore D. NF-kappa B: a pleiotropic mediator of inducible and tissue-specific gene control. Cell. (1989) 58:227–9. 10.1016/0092-8674(89)90833-7 2665943

[5] Liu T Zhang L Joo D Sun S-C. NF-κB signaling in inflammation. Signal Transduct Target Ther. (2017) 2:17023. 10.1038/sigtrans.2017.23 29158945 PMC5661633

[6] Medzhitov R Horng T. Transcriptional control of the inflammatory response. Nat Rev Immunol. (2009) 9:692–703. 10.1038/nri2634 19859064

[7] Cildir G Low KC Tergaonkar V. Noncanonical NF-κB signaling in health and disease. Trends Mol Med. (2016) 22:414–29. 10.1016/j.molmed.2016.03.002 27068135

[8] Kaileh M Sen R. NF-κB function in B lymphocytes. Immunol Rev. (2012) 246:254–71. 10.1111/j.1600-065X.2012.01106.x 22435560

[9] Alcamo E Hacohen N Schulte LC Rennert PD Hynes RO Baltimore D. Requirement for the NF-kappaB family member RelA in the development of secondary lymphoid organs. J Exp Med. (2002) 195:233–44. 10.1084/jem.20011885 11805150 PMC2193608

[10] Courtine E Cagnard N Mazzolini J Antona M Pène F Fitting C. Combined loss of cRel/p50 subunits of NF-κB leads to impaired innate host response in sepsis. Innate Immun. (2012) 18:753–63. 10.1177/1753425912440296 22408080

[11] Grigoriadis G Zhan Y Grumont RJ Metcalf D Handman E Cheers C. The Rel subunit of NF-kappaB-like transcription factors is a positive and negative regulator of macrophage gene expression: distinct roles for Rel in different macrophage populations. EMBO J. (1996) 15:7099–107. 10.1002/j.1460-2075.1996.tb01101.x 9003785 PMC452535

[12] Sanjabi S Hoffmann A Liou HC Baltimore D Smale ST. Selective requirement for c-Rel during IL-12 P40 gene induction in macrophages. Proc Natl Acad Sci. USA. (2000) 97:12705–10. 10.1073/pnas.230436397 11058167 PMC18828

[13] Wang X Wang J Zheng H Xie M Hopewell EL Albrecht RA. Differential requirement for the IKKβ/NF-κB signaling module in regulating TLR- versus RLR-induced type 1 IFN expression in dendritic cells. J Immunol. (2014) 193:2538–45. 10.4049/jimmunol.1400675 25057006 PMC4134964

[14] Werner SL Barken D Hoffmann A. Stimulus specificity of gene expression programs determined by temporal control of IKK activity. Science. (2005) 309:1857–61. 10.1126/science.1113319 16166517

[15] González-Crespo S Levine M. Related target enhancers for dorsal and NF-kappa B signaling pathways. Science. (1994) 264:255–8. 10.1126/science.8146656 8146656

[16] Leung TH Hoffmann A Baltimore D. One nucleotide in a κB site can determine cofactor specificity for NF-κB dimers. Cell. (2004) 118:453–64. 10.1016/j.cell.2004.08.007 15315758

[17] Savinova OV Hoffmann A Ghosh G. The Nfkb1 and Nfkb2 proteins p105 and p100 function as the core of high-molecular-weight heterogeneous complexes. Mol Cell. (2009) 34:591–602. 10.1016/j.molcel.2009.04.033 19524538 PMC4167889

[18] Ghashghaeinia M Toulany M Saki M Rodemann HP Mrowietz U Lang F. Potential roles of the NFκB and glutathione pathways in mature human erythrocytes. Cell Mol Biol Lett. (2012) 17:11–20. 10.2478/s11658-011-0032-x 22105338 PMC6275705

[19] Zambrano S Bianchi ME Agresti A. High-throughput analysis of NF-κB dynamics in single cells reveals basal nuclear localization of NF-κB and spontaneous activation of oscillations. PLoS ONE. (2014) 9:e90104. 10.1371/journal.pone.0090104 24595030 PMC3942427

[20] Zarnegar BJ Wang Y Mahoney DJ Dempsey PW Cheung HH He J. Noncanonical NF-kappaB activation requires coordinated assembly of a regulatory complex of the adaptors cIAP1, cIAP2, TRAF2 and TRAF3 and the kinase NIK. Nat Immunol. (2008) 9:1371–8. 10.1038/ni.1676 18997794 PMC2676931

[21] Schröfelbauer B Polley S Behar M Ghosh G Hoffmann A. NEMO ensures signaling specificity of the pleiotropic IKKβ by directing its kinase activity toward IκBα. Mol Cell. (2012) 47:111–21. 10.1016/j.molcel.2012.04.020 22633953 PMC3398199

[22] Kanarek N Ben-Neriah Y. Regulation of NF-κB by ubiquitination and degradation of the IκBs. Immunol Rev. (2012) 246:77–94. 10.1111/j.1600-065X.2012.01098.x 22435548

[23] Osborn L Kunkel S Nabel GJ. Tumor necrosis factor alpha and interleukin 1 stimulate the human immunodeficiency virus enhancer by activation of the nuclear factor kappa B. Proc Natl Acad Sci. USA. (1989) 86:2336–40. 10.1073/pnas.86.7.2336 2494664 PMC286907

[24] Medzhitov R Preston-Hurlburt P Janeway CA. A human homologue of the *Drosophila* toll protein signals activation of adaptive immunity. Nature. (1997) 388:394–7. 10.1038/41131 9237759

[25] Seth RB Sun L Ea C-K Chen ZJ. Identification and characterization of MAVS, a mitochondrial antiviral signaling protein that activates NF-kappaB and IRF 3. Cell. (2005) 122:669–82. 10.1016/j.cell.2005.08.012 16125763

[26] Fang R Wang C Jiang Q Lv M Gao P Yu X. NEMO-IKKβ are essential for IRF3 and NF-κB activation in the cGAS-STING pathway. J Immunol. (2017) 199:3222–33. 10.4049/jimmunol.1700699 28939760

[27] Schreck R Bevec D Dukor P Baeuerle PA Chedid L Bahr GM. Selection of a muramyl peptide based on its lack of activation of nuclear factor-kappa B as a potential adjuvant for AIDS vaccines. Clin Exp Immunol. (1992) 90:188–93. 10.1111/j.1365-2249.1992.tb07926.x 1424273 PMC1554598

[28] Coudronniere N Villalba M Englund N Altman A. NF-kappa B activation induced by T cell receptor/CD28 costimulation is mediated by protein kinase C-theta. Proc Natl Acad Sci USA. (2000) 97:3394–9. 10.1073/pnas.060028097 10716728 PMC16250

[29] Petro JB Rahman SM Ballard DW Khan WN. Bruton's tyrosine kinase is required for activation of IkappaB kinase and nuclear factor kappaB in response to B cell receptor engagement. J Exp Med. (2000) 191:1745–54. 10.1084/jem.191.10.1745 10811867 PMC2193161

[30] Kumar H Kawai T Akira S. Toll-like receptors and innate immunity. Biochem Biophys Res Commun. (2009) 388:621–5. 10.1016/j.bbrc.2009.08.062 19686699

[31] Kagan JC Su T Horng T Chow A Akira S Medzhitov R. TRAM couples endocytosis of Toll-like receptor 4 to the induction of interferon-β. Nat Immunol. (2008) 9:361–8. 10.1038/ni1569 18297073 PMC4112825

[32] Toshchakov V Jones BW Perera P-Y Thomas K Cody MJ Zhang S TLR4, but not TLR2, mediates IFN-beta-induced STAT1alpha/beta-dependent gene expression in macrophages. Nat Immunol. (2002) 3:392–8. 10.1038/ni774 11896392

[33] Park SH Kang K Giannopoulou E Qiao Y Kang K Kim G. Type I interferons and the cytokine TNF cooperatively reprogram the macrophage epigenome to promote inflammatory activation. Nat Immunol. (2017) 18:1104–16. 10.1038/ni.3818 28825701 PMC5605457

[34] Borghini L Lu J Hibberd M Davila S. Variation in genome-wide NF-κB RELA binding sites upon microbial stimuli and identification of a virus response profile. J Immunol. (2018) 201:1295–305. 10.4049/jimmunol.1800246 29959281

[35] Ashall L Horton CA Nelson DE Paszek P Harper CV Sillitoe K. Pulsatile stimulation determines timing and specificity of NF-kappaB-dependent transcription. Science. (2009) 324:242–6. 10.1126/science.1164860 19359585 PMC2785900

[36] Heltberg M Kellogg RA Krishna S Tay S Jensen MH. Noise induces hopping between NF-andkappa;B entrainment modes. Cell Syst. (2016) 3:532–9.e3. 10.1016/j.cels.2016.11.014 28009264 PMC5783698

[37] Kellogg RA Tian C Lipniacki T Quake SR Tay S. Digital signaling decouples activation probability and population heterogeneity. Elife. (2015) 4:e08931. 10.7554/eLife.08931 26488364 PMC4608393

[38] Mauro C Pacifico F Lavorgna A Mellone S Iannetti A Acquaviva R. ABIN-1 binds to NEMO/IKKγ and co-operates with A20 in inhibiting NF-κB. J Biol Chem. (2006) 281:18482–8. 10.1074/jbc.M601502200 16684768

[39] Garg AV Ahmed M Vallejo AN Ma A Gaffen SL. The deubiquitinase A20 mediates feedback inhibition of interleukin-17 receptor signaling. Sci Signal. (2013) 6:ra44. 10.1126/scisignal.2003699 23737552 PMC4028484

[40] Hughey JJ Gutschow MV Bajar BT Covert MW. Single-cell variation leads to population invariance in NF-κB signaling dynamics. Mol Biol Cell. (2015) 26:583–90. 10.1091/mbc.E14-08-1267 25473117 PMC4310747

[41] Lane K Van Valen D DeFelice MM Macklin DN Kudo T Jaimovich A. Measuring signaling and RNA-seq in the same cell links gene expression to dynamic patterns of NF-κB activation. Cell Syst. (2017) 4:458–69.e5. 10.1016/j.cels.2017.03.010 28396000 PMC6748049

[42] Tay S Hughey JJ Lee TK Lipniacki T Quake SR Covert MW. Single-cell NF-κB dynamics reveal digital activation and analogue information processing. Nature. (2010) 466:267–71. 10.1038/nature09145 20581820 PMC3105528

[43] Basak S Behar M Hoffmann A. Lessons from mathematically modeling the NF-κB pathway. Immunol Rev. (2012) 246:221–38. 10.1111/j.1600-065X.2011.01092.x 22435558 PMC3343698

[44] Ankers JM Awais R Jones NA Boyd J Ryan S Adamson AD. Dynamic NF-κB and E2F interactions control the priority and timing of inflammatory signalling and cell proliferation. Elife. (2016) 5:375. 10.7554/eLife.10473 27185527 PMC4869934

[45] Muxel SM Laranjeira-Silva MF Carvalho-Sousa CE Floeter-Winter LM Markus RP. The RelA/cRel nuclear factor-κB (NF-κB) dimer, crucial for inflammation resolution, mediates the transcription of the key enzyme in melatonin synthesis in RAW 264.7 macrophages. J Pineal Res. (2016) 60:394–404. 10.1111/jpi.12321 26887983

[46] Litvak V Ramsey SA Rust AG Zak DE Kennedy KA Lampano AE. Function of C/EBPδ in a regulatory circuit that discriminates between transient and persistent TLR4-induced signals. Nat Immunol. (2009) 10:437–43. 10.1038/ni.1721 19270711 PMC2780024

[47] Caldwell AB Cheng Z Vargas JD Birnbaum HA Hoffmann A. Network dynamics determine the autocrine and paracrine signaling functions of TNF. Genes Dev. (2014) 28:2120–33. 10.1101/gad.244749.114 25274725 PMC4180974

[48] Cheng Z Taylor B Ourthiague DR Hoffmann A. Distinct single-cell signaling characteristics are conferred by the MyD88 and TRIF pathways during TLR4 activation. Sci Signal. (2015) 8:ra69. 10.1126/scisignal.aaa5208 26175492 PMC6764925

[49] Sakai J Cammarota E Wright JA Cicuta P Gottschalk RA Li N. Lipopolysaccharide-induced NF-κB nuclear translocation is primarily dependent on MyD88, but TNFα expression requires TRIF and MyD88. Sci Rep. (2017) 7:1428. 10.1038/s41598-017-01600-y 28469251 PMC5431130

[50] Sung MH Li N Lao Q Gottschalk RA Hager GL Fraser IDC Switching of the relative dominance between feedback mechanisms in lipopolysaccharide-induced NF- B signaling. Sci Signal. (2014) 7:ra6 10.1126/scisignal.2004764 24425788 PMC5381725

[51] Covert MW Leung TH Gaston JE Baltimore D. Achieving stability of lipopolysaccharide-induced NF-kappaB activation. Science. (2005) 309:1854–7. 10.1126/science.1112304 16166516

[52] Kawai T Adachi O Ogawa T Takeda K Akira S. Unresponsiveness of MyD88-deficient mice to endotoxin. Immunity. (1999) 11:115–22. 10.1016/S1074-7613(00)80086-2 10435584

[53] Kellogg RA Tian C Etzrodt M Tay S. Cellular decision making by non-integrative processing of TLR inputs. Cell Rep. (2017) 19:125–35. 10.1016/j.celrep.2017.03.027 28380352 PMC5766323

[54] Sato S Sugiyama M Yamamoto M Watanabe Y Kawai T Takeda K. Toll/IL-1 receptor domain-containing adaptor inducing IFN-beta (TRIF) associates with TNF receptor-associated factor 6 and TANK-binding kinase 1, and activates two distinct transcription factors, NF-kappa B and IFN-regulatory factor-3, in the Toll-like receptor signaling. J Immunol. (2003) 171:4304–10. 10.4049/jimmunol.171.8.4304 14530355

[55] Oh K-S Gottschalk RA Lounsbury NW Sun J Dorrington MG Baek S. Dual roles for ikaros in regulation of macrophage chromatin state and inflammatory gene expression. J Immunol. (2018) 201:757–71. 10.4049/jimmunol.1800158 29898962 PMC6069956

[56] Parnas O Jovanovic M Eisenhaure TM Herbst RH Dixit A Ye CJ. A Genome-wide CRISPR screen in primary immune cell*s* to dissect regulatory networks. Cell. (2015) 162:675–86. 10.1016/j.cell.2015.06.059 26189680 PMC4522370

[57] Stein B Baldwin AS Ballard DW Greene WC Angel P Herrlich P. Cross-coupling of the NF-kappa B p65 and Fos/Jun transcription factors produces potentiated biological function. EMBO J. (1993) 12:3879–91. 10.1002/j.1460-2075.1993.tb06066.x 8404856 PMC413671

[58] Nelson DE Ihekwaba AEC Elliott M Johnson JR Gibney CA Foreman BE. Oscillations in NF-kappaB signaling control the dynamics of gene expression. Science. (2004) 306:704–8. 10.1126/science.1099962 15499023

[59] Sung M-H Salvatore L De Lorenzi R Indrawan A Pasparakis M Hager GL. Sustained oscillations of NF-kappaB produce distinct genome scanning and gene expression profiles. PLoS ONE. (2009) 4:e7163. 10.1371/journal.pone.0007163 19787057 PMC2747007

[60] Zambrano S De Toma I Piffer A Bianchi ME Agresti A. NF-κB oscillations translate into functionally related patterns of gene expression. Elife. (2016) 5:e09100. 10.7554/eLife.09100 26765569 PMC4798970

[61] Barboric M Nissen RM Kanazawa S Jabrane-Ferrat N Peterlin BM. NF-kappaB binds P-TEFb to stimulate transcriptional elongation by RNA polymerase II. Mol Cell. (2001) 8:327–37. 10.1016/S1097-2765(01)00314-8 11545735

[62] Hargreaves DC Horng T Medzhitov R. Control of inducible gene expression by signal-dependent transcriptional elongation. Cell. (2009) 138:129–45. 10.1016/j.cell.2009.05.047 19596240 PMC2828818

[63] Brown JD Lin CY Duan Q Griffin G Federation A Paranal RM. NF-κB directs dynamic super enhancer formation in inflammation and atherogenesis. Mol Cell. (2014) 56:219–31. 10.1016/j.molcel.2014.08.024 25263595 PMC4224636

[64] Lee TK Denny EM Sanghvi JC Gaston JE Maynard ND Hughey JJ. A noisy paracrine signal determines the cellular NF-kappaB response to lipopolysaccharide. Sci Signal. (2009) 2:ra65. 10.1126/scisignal.2000599 19843957 PMC2778577

[65] Liu Q Zhu Y Yong WK Sze NSK Tan NS Ding JL. Cutting edge: synchronization of IRF1, JunB, and C/EBPβ activities during TLR3-TLR7 cross-talk orchestrates timely cytokine synergy in the proinflammatory response. J Immunol. (2015) 195:801–5. 10.4049/jimmunol.1402358 26109639 PMC4505950

[66] Meier A Alter G Frahm N Sidhu H Li B Bagchi A. MyD88-dependent immune activation mediated by human immunodeficiency virus type 1-encoded Toll-like receptor ligands. J Virol. (2007) 81:8180–91. 10.1128/JVI.00421-07 17507480 PMC1951290

[67] Napolitani G Rinaldi A Bertoni F Sallusto F Lanzavecchia A. Selected toll-like receptor agonist combinations synergistically trigger a T helper type 1-polarizing program in dendritic cells. Nat Immunol. (2005) 6:769–76. 10.1038/ni1223 15995707 PMC3760217

[68] Suet Ting Tan R Lin B Liu Q Tucker-Kellogg L Ho B Leung BPL. The synergy in cytokine production through MyD88-TRIF pathways is co-ordinated with ERK phosphorylation in macrophages. Immunol Cell Biol. (2013) 91:377–87. 10.1038/icb.2013.13 23567895

[69] Lin B Dutta B Fraser IDC. Systematic investigation of multi-TLR sensing identifies regulators of sustained gene activation in macrophages. Cell Syst. (2017) 5:25–37.e3. 10.1016/j.cels.2017.06.014 28750197 PMC5584636

[70] Hodgson A Wan F. Interference with nuclear factor kappaB signaling pathway by pathogen-encoded proteases: global and selective inhibition. Mol Microbiol. (2016) 99:439–52. 10.1111/mmi.13245 26449378 PMC5003429

[71] Li K Foy E Ferreon JC Nakamura M Ferreon ACM Ikeda M. Immune evasion by hepatitis C virus NS3/4A protease-mediated cleavage of the Toll-like receptor 3 adaptor protein TRIF. Proc Natl Acad Sci USA. (2005) 102:2992–7. 10.1073/pnas.0408824102 15710891 PMC548795

[72] Novikova L Czymmeck N Deuretzbacher A Buck F Richter K Weber ANR. Cell death triggered by *Yersinia enterocolitica* identifies processing of the proinflammatory signal adapter MyD88 as a general event in the execution of apoptosis. J Immunol. (2014) 192:1209–19. 10.4049/jimmunol.1203464 24363429

[73] Xiang Z Li L Lei X Zhou H Zhou Z He B. Enterovirus 68 3C protease cleaves TRIF to attenuate antiviral responses mediated by Toll-like receptor 3. J Virol. (2014) 88:6650–9. 10.1128/JVI.03138-13 24672048 PMC4054379

[74] Baruch K Gur-Arie L Nadler C Koby S Yerushalmi G Ben-Neriah Y. Metalloprotease type III effectors that specifically cleave JNK and NF-κB. EMBO J. (2011) 30:221–31. 10.1038/emboj.2010.297 21113130 PMC3020117

[75] Mühlen S Ruchaud-Sparagano M-H Kenny B. Proteasome-independent degradation of canonical NFkappaB complex components by the NleC protein of pathogenic *Escherichia coli*. J Biol Chem. (2011) 286:5100–7. 10.1074/jbc.M110.172254 21148319 PMC3037621

[76] Pearson JS Riedmaier P Marchès O Frankel G Hartland EL. A type III effector protease NleC from enteropathogenic *Escherichia coli* targets NF-κB for degradation. Mol Microbiol. (2011) 80:219–30. 10.1111/j.1365-2958.2011.07568.x 21306441 PMC3178796

[77] Yen H Ooka T Iguchi A Hayashi T Sugimoto N Tobe T. NleC, a type III secretion protease, compromises NF-κB activation by targeting p65/RelA. PLoS Pathog. (2010) 6:e1001231. 10.1371/journal.ppat.1001231 21187904 PMC3002990

[78] Roy CR Mocarski ES. Pathogen subversion of cell-intrinsic innate immunity. Nat Immunol. (2007) 8:1179–87. 10.1038/ni1528 17952043

[79] Gottschalk RA Martins AJ Angermann BR Dutta B Ng CE Uderhardt S. Distinct NF-κB and MAPK activation thresholds uncouple steady-state microbe sensing from anti-pathogen inflammatory responses. Cell Syst. (2016) 2:378–90. 10.1016/j.cels.2016.04.016 27237739 PMC4919147

[80] Ramirez-Carrozzi VR Braas D Bhatt DM Cheng CS Hong C Doty KR. A unifying model for the selective regulation of inducible transcription by CpG islands and nucleosome remodeling. Cell. (2009) 138:114–28. 10.1016/j.cell.2009.04.020 19596239 PMC2712736

[81] Kline KA Bowdish DME. Infection in an aging population. Curr Opin Microbiol. (2015) 29:63–7. 10.1016/j.mib.2015.11.003 26673958

[82] Puchta A Naidoo A Verschoor CP Loukov D Thevaranjan N Mandur TS. TNF drives monocyte dysfunction with age and results in impaired anti-pneumococcal immunity. PLoS Pathog. (2016) 12:e1005368. 10.1371/journal.ppat.1005368 26766566 PMC4713203

[83] Thevaranjan N Puchta A Schulz C Naidoo A Szamosi JC Verschoor CP. Age-associated microbial dysbiosis promotes intestinal permeability, systemic inflammation, and macrophage dysfunction. Cell Host Microbe. (2017) 21:455–66.e4. 10.1016/j.chom.2017.03.002 28407483 PMC5392495

[84] Barbet G Sander LE Geswell M Leonardi I Cerutti A Iliev I. Sensing microbial viability through bacterial RNA augments T follicular helper cell and antibody responses. Immunity. (2018) 48:584–98.e5. 10.1016/j.immuni.2018.02.015 29548673 PMC5924674

[85] Moretti J Roy S Bozec D Martinez J Chapman JR Ueberheide B. STING senses microbial viability to orchestrate stress-mediated autophagy of the endoplasmic reticulum. Cell. (2017) 171:809–16.e13. 10.1016/j.cell.2017.09.034 29056340 PMC5811766

[86] Mourao-Sa D Roy S Blander JM. Vita-PAMPs: signatures of microbial viability. Adv Exp Med Biol. (2013) 785:1–8. 10.1007/978-1-4614-6217-0_1 23456832

[87] Sander LE Davis MJ Boekschoten MV Amsen D Dascher CC Ryffel B. Detection of prokaryotic mRNA signifies microbial viability and promotes immunity. Nature. (2011) 474:385–9. 10.1038/nature10072 21602824 PMC3289942

[88] Bagnall J Boddington C England H Brignall R Downton P Alsoufi Z. Quantitative analysis of competitive cytokine signaling predicts tissue thresholds for the propagation of macrophage activation. Sci Signal. (2018) 11:eaaf3998. 10.1126/scisignal.aaf3998 30042130

[89] Boone DL Turer EE Lee EG Ahmad R-C Wheeler MT Tsui C. The ubiquitin-modifying enzyme A20 is required for termination of Toll-like receptor responses. Nat Immunol. (2004) 5:1052–60. 10.1038/ni1110 15334086

[90] Lee REC Walker SR Savery K Frank DA Gaudet S. Fold Change of nuclear NF-andkappa;B Determines TNF-induced transcription in single cells. Mol Cell. (2014) 53:867–79. 10.1016/j.molcel.2014.01.026 24530305 PMC3977799

[91] Shih VF-S Davis-Turak J Macal M Huang JQ Ponomarenko J Kearns JD. Control of RelB during dendritic cell activation integrates canonical and noncanonical NF-κB pathways. Nat Immunol. (2012) 13:1162–70. 10.1038/ni.2446 23086447 PMC3634611

[92] Li M Zhang X Zheng X Lian D Zhang Z-X Ge W. Immune modulation and tolerance induction by RelB-silenced dendritic cells through RNA interference. J Immunol. (2007) 178:5480–7. 10.4049/jimmunol.178.9.5480 17442929

[93] Nair PM Starkey MR Haw TJ Ruscher R Liu G Maradana MR. RelB-deficient dendritic cells promote the development of spontaneous allergic airway inflammation. Am J Respir Cell Mol Biol. (2018) 58:352–65. 10.1165/rcmb.2017-0242OC 28960101

[94] Ammon C Mondal K Andreesen R Krause SW. Differential expression of the transcription factor NF-kappaB during human mononuclear phagocyte differentiation to macrophages and dendritic cells. Biochem Biophys Res Commun. (2000) 268:99–105. 10.1006/bbrc.1999.2083 10652220

[95] Kobayashi T Walsh PT Walsh MC Speirs KM Chiffoleau E King CG. TRAF6 is a critical factor for dendritic cell maturation and development. Immunity. (2003) 19:353–63. 10.1016/S1074-7613(03)00230-9 14499111

[96] McDonald PP Bald A Cassatella MA. Activation of the NF-kappaB pathway by inflammatory stimuli in human neutrophils. Blood. (1997) 89:3421–33. 9129050

[97] Castro-Alcaraz S Miskolci V Kalasapudi B Davidson D Vancurova I NF-κB regulation in human neutrophils by nuclear IκBα: correlation to apoptosis. J Immunol. (2002) 169:3947–53. 10.4049/jimmunol.169.7.3947 12244195

[98] Regot S Hughey JJ Bajar BT Carrasco S Covert MW. High-sensitivity measurements of multiple kinase activities in live single cell*s*. Cell. (2014) 157:1724–34. 10.1016/j.cell.2014.04.039 24949979 PMC4097317

[99] Carlotti F Dower SK Qwarnstrom EE Dynamic shuttling of nuclear factor κB between the nucleus and cytoplasm as a consequence of inhibitor dissociation. J Biol. Chem. (2000) 275:41028–34. 10.1074/jbc.M006179200 11024020

[100] Hoffmann A Levchenko A Scott ML Baltimore D. The IkappaB-NF-kappaB signaling module: temporal control and selective gene activation. Science. (2002) 298:1241–5. 10.1126/science.1071914 12424381

[101] Kearns JD Basak S Werner SL Huang CS Hoffmann A. IκBε provides negative feedback to control NF-κB oscillations, signaling dynamics, and inflammatory gene expression. J Cell Biol. (2006) 173:659–64. 10.1083/jcb.200510155 16735576 PMC2063883

[102] Koh G Lee D-Y. Mathematical modeling and sensitivity analysis of the integrated TNFα-mediated apoptotic pathway for identifying key regulators. Comput Biol Med. (2011) 41:512–28. 10.1016/j.compbiomed.2011.04.017 21632045

[103] Peng SC Wong DSH Tung KC Chen YY Chao CC Peng CH. Computational modeling with forward and reverse engineering links signaling network and genomic regulatory responses: NF-kappaB signaling-induced gene expression responses in inflammation. BMC Bioinformatics. (2010) 11:308. 10.1186/1471-2105-11-308 20529327 PMC2889938

[104] Werner SL Kearns JD Zadorozhnaya V Lynch C O'Dea E Boldin MP. Encoding NF-kappaB temporal control in response to TNF: distinct roles for the negative regulators IkappaBalpha and A20. Genes Dev. (2008) 22:2093–101. 10.1101/gad.1680708 18676814 PMC2492747

[105] Yde P Mengel B Jensen MH Krishna S Trusina A. Modeling the NF-κB mediated inflammatory response predicts cytokine waves in tissue. BMC Syst Biol. (2011) 5:115. 10.1186/1752-0509-5-115 21771307 PMC3152534

[106] Finn JD Smith AR Patel MC Shaw L Youniss MR van Heteren J. A Single administration of CRISPR/Cas9 lipid nanoparticles achieves robust and persistent inandnbsp;*Vivo* genome editing. Cell Rep. (2018) 22:2227–35. 10.1016/j.celrep.2018.02.014 29490262

[107] Helmchen F Denk W. Deep tissue two-photon microscopy. Nat Meth. (2005) 2:932–40. 10.1038/nmeth818 16299478

[108] Koechlein CS Harris JR Lee TK Weeks J Fox RG Zimdahl B. High-resolution imaging and computational analysis of haematopoietic cell dynamics *in vivo*. Nat Commun. (2016) 7:12169. 10.1038/ncomms12169 27425143 PMC4960315

